# Unveiling the Impact of Processing Methods on In Vitro Protein Digestibility: A Focus on Highland Barley and Its Application in Wine Production

**DOI:** 10.3390/foods14122020

**Published:** 2025-06-07

**Authors:** Mengchuan He, Xinjing Zhou, András Koris, Bingyu Chen, Xuchun Zhu, Feiyue Ren, Hongzhi Liu

**Affiliations:** 1 Key Laboratory of Geriatric Nutrition and Health, Ministry of Education, School of Food and Health, Beijing Technology & Business University (BTBU), Beijing 100048, China; 13831340605@163.com (M.H.); 2431032112@st.btbu.edu.cn (X.Z.); bychen313@gmail.com (B.C.); xxcy08@163.com (X.Z.); 2 Department of Food Process Engineering, Institute of Food Science and Technology, Hungarian University of Agriculture and Life Sciences, Ménesi út 44, HU-1118 Budapest, Hungary; koris.andras@uni-mate.hu

**Keywords:** highland barley protein, in vitro protein digestibility, in vitro digestive model, highland barley wine, processing method

## Abstract

As one of the indispensable components of the human body, proteins not only participate in the construction of tissues, but also play an important role in maintaining the metabolic balance of the organism. However, in some protein sources, factors such as complex protein structures and the presence of anti-nutritional compounds reduce bioavailability. Processing methods can significantly enhance protein digestibility by overcoming these limitations. This review focuses on highland barley proteins and their application in winemaking. It summarises their composition, digestibility and functional properties. It also summarises and evaluates in vitro digestion models suitable for in vitro digestion of highland barley proteins. Then, this review investigates in depth the effects of different processing methods—including thermal treatments, enzymatic hydrolysis, and microbial fermentation—on the in vitro digestibility of highland barley proteins and their mechanisms during winemaking. It was shown that these processing methods achieve different degrees of in vitro protein digestibility improvement by altering the structure of highland barley proteins and altering the interactions with other substances, achieving efficient utilisation of high-quality highland barley protein resources. Finally, this review provides a prospect which opens up new ideas for the efficient utilisation of proteins in food processing in the future.

## 1. Introduction

Protein, one of the seven essential nutrients, plays a crucial structural and functional role in supporting human growth and physiological processes [1]. Dietary proteins can be enzymatically converted to amino acids during digestion. In addition to prolonging satiety, they can provide the raw material base for protein biosynthesis in the body [2,3]. These amino acids participate in a wide range of biochemical activities, including enzymatic catalysis, cellular motility, hormonal signalling, immune responses, structural support, and nutrient transport and storage.

Highland barley, also known as hulless barley, genus Barley of the wheat family of the gramineae, is a highland cereal crop that is drought-resistant, barren-resistant, cold-resistant, and stress-resistant, and is mainly produced in China’s Tibet, Qinghai, Sichuan, and other places. Among them, Tibet and Qinghai accounted for almost 90% of the total highland barley production in 2018. There are about 501 varieties of highland barley in the Tibet Autonomous Region [4]. Recently, highland barley protein has received a lot of attention as a high-quality protein. The protein content of highland barley ranges from 6.35 to 23.4%, with about 30% of essential amino acids [4]. However, compared with wheat, highland barley protein has poorer protein aggregation behaviour and protein pasting characteristics, so its application as a staple food in the processing of flour products has been limited, and it is currently only processed into bread, noodles, and cookies [5]. In contrast, highland barley proteins show excellent properties in winemaking [6]. As a characteristic traditional drink of the Tibetan Plateau, highland barley wine enjoys a high reputation among consumer groups for its moderate alcohol content (5–10% vol), unique flavour characteristics, rich nutrients, and sweet and sour taste qualities [7].

It is essential to evaluate protein nutritional value accurately. It depends not only on amino acid composition and structure, but more critically on digestibility within the gastrointestinal tract (GI tract) [8]. Protein digestibility reflects the proportion of ingested protein that is hydrolysed and absorbed, representing the bioavailability of amino acids from dietary sources [9]. There are two main digestion models for studying protein digestibility, in vivo and in vitro [10]. In vivo modelling usually involves feeding proteins to test animals, then scratching them and seeing how much protein is digested at the end of the small intestine, which in turn calculates protein digestibility. However, this method takes more time and money and has ethical implications [11]. The physiological environment and digestive processes of animals also tend to differ from those of the human body and vary greatly from one individual to another. Therefore, in vitro digestion models have become an important tool for studying in vivo food digestion [12].

Highland barley protein may have limited physical and chemical utilisation during digestion and absorption due to its structure and the presence of anti-nutritional factors that inhibit digestion and absorption, such as lectins, tannins, phytates, and protease inhibitors [13]. Thus, processing can alter the digestibility [14,15]. Focusing on the application of highland barley proteins in winemaking, this paper explores the mechanisms by which key processing methods affect the in vitro protein digestibility (IVPD) of highland barley. It is shown that thermal processing during highland barley wine brewing loosens the protein structure and increases the binding sites for proteases. The addition of exogenous proteases promoted protein hydrolysis. At the same time, the addition of phytase promoted the hydrolysis of phytic acid, thus reducing the anti-nutritional factors in highland barley protein and improving protein digestibility [16]. Microorganisms also secrete a large number of enzymes, including proteases that break down large proteins into small proteins, increasing protein content and facilitating digestion and absorption [17]. These processing methods significantly improved the digestibility of highland barley proteins, thus allowing them to be utilised efficiently, which is important for the efficient use of protein resources.

## 2. Nutritional and Functional Properties of Highland Barley Protein

### 2.1. Nutritional Value of Highland Barley Protein

#### 2.1.1. Composition of Highland Barley Protein

The protein content of highland barley is 6.35 to 23.4%, twice that of rice and 1.5 times that of wheat, sorghum, and maize [4,18]. This makes it a high-quality plant protein resource. Highland barley protein contains all 18 amino acids, including 8 essential ones. The content of essential amino acids is approximately 27.11 mg/g DW (Table 1).

Based on the amino acid ratio coefficient (RC), 91.67% of the highland barley materials had lysine as the first limiting amino acid, while some materials had isoleucine, methionine, or cysteine as the first limiting amino acid. This pattern is similar to most cereal proteins. However, the relatively high lysine content of highland barley protein, up to 3.64 mg/g DW, is a distinct advantage [19].

The molecular weight of highland barley proteins ranges from 10 to 95 kDa [22]. According to solubility, these proteins are categorised into four main types: albumins, globulins, hordeins, and glutelins. Their proportions, molecular weights, and key characteristics are summarised in Table 2 [4,23,24,25,26].

#### 2.1.2. Digestibility of Highland Barley Protein and Its Influencing Factors

The nutritional value of proteins is not only related to the types of amino acids they contain, but also closely associated with their absorption efficiency in the digestive tract [8,27]. Protein digestibility reflects the proportion of ingested protein that is hydrolysed and absorbed, indicating the bioavailability of amino acids from dietary sources [9].

The IVPD of highland barley is approximately 90.02 ± 2.24%, which is higher than that of buckwheat (74.66%), barley (74.98%), wheat (85.47%), and quinoa (85.79%) [22]. This suggests that highland barley protein is relatively easy to digest, although there is still room for improvement. Three main factors influence the digestibility of highland barley protein: 1. Protein molecular structure, including amino acid composition and folding pattern. 2. Co-existing substances, such as anti-nutritional factors, starch, and polysaccharides (e.g., pectin and cellulose). 3. Digestive conditions, including pH, temperature, ionic strength, digestion time, and the type and concentration of enzymes.

The amino acid profile strongly affects protein hydrolysis because peptidases are specific to peptide bonds near certain amino acids. For instance, proline resists enzymatic hydrolysis, making proline-rich proteins harder to digest [28]. Highland barley protein contains about 9.73 mg/g (DW) proline—which is higher than in most cereals [19].

Additionally, lysine has a blocked ε-amino group, making it less bioavailable and reducing digestibility [29]. The protein structure also plays a crucial role. Disulfide cross-links stabilise the protein, making it more resistant to enzymatic breakdown [29]. The disulfide bond and total sulfhydryl content of glutenin, the largest proportion of highland barley protein, were 10.3779 μmol/g and 88.2799 μmol/g, respectively [24]. These free sulfhydryl groups can promote further disulfide bond formation, further stabilising the protein.

Tightly folded structures or protein aggregation can limit enzyme access to peptide chains, thus slowing hydrolysis. In highland barley glutenin, the secondary structure is dominated by β-turns (34.31%) and β-sheets (35.63%). In contrast, the content of α-helices (11.82%) and random coils (11.76%) is relatively low, indicating a more stable, compact structure [26].

The presence of anti-nutritional factors can limit protein digestibility [30]. Major anti-nutritional factors in highland barley include protease inhibitors, tannins, and phytate. Protease inhibitors reduce IVPD by inactivating trypsin (the most important peptidase in the GI tract) [28,30]. Tannins are phenolic substances that interact with proteins and minerals in the aqueous environment to form precipitates, thus reducing the hydrolytic activity of proteins [31]. Phytate, on the one hand, reduces protein digestibility by competing for activity with peptidases. On the other hand, due to its high density of negatively charged phosphate groups, phytate has a strong metal-chelating activity and forms very stable complexes with mineral ions, which makes it unavailable for intestinal ingestion [32,33]. Reduced bioavailability of minerals interferes with protein digestibility [34]. Anti-nutritional factors are found more often in plant proteins than in proteins of animal origin. Also, the rigid cell walls or seed coat of plants can make digestive enzymes less accessible [29]. Plant proteins are often embedded within cellular matrices in the form of discrete proteasomes and exhibit relatively low solubility. Additionally, the low permeability of plant cell walls and the protective nature of seed coats further restrict the accessibility of digestive enzymes [29]. Consequently, plant proteins typically require additional processing to modify their structure and enhance digestibility.

The presence of polysaccharides also affects protein digestion. Plant cell walls contain several different proteins, most of which are glycosylated [35]. These glycoproteins are highly resistant to most proteases, especially when oligoarabinose side chains remain attached. Thus, binding between proteins and grain cell walls may reduce protein digestibility by reducing enzyme accessibility or forming indigestible complexes [36]. Starch granules and proteasomes are closely related to each other. Most polygonal, tightly packed starch granules are surrounded by a large number of spherical proteasomes embedded in the protein matrix, and this close association may make protein digestion difficult [35].

pH, temperature, and ionic strength all alter protein digestibility by affecting protease activity. The type of enzyme also affects protein digestibility. The use of pepsin at levels greater than 90% was considered as a digestive enzyme, compared to trypsin, which has a much lower IVPD (less than 37%) [37]. Therefore, the properties of the enzymes used need to be considered when constructing an in vitro digestive system.

### 2.2. Functional Properties of Highland Barley Protein

**Figure 1:**
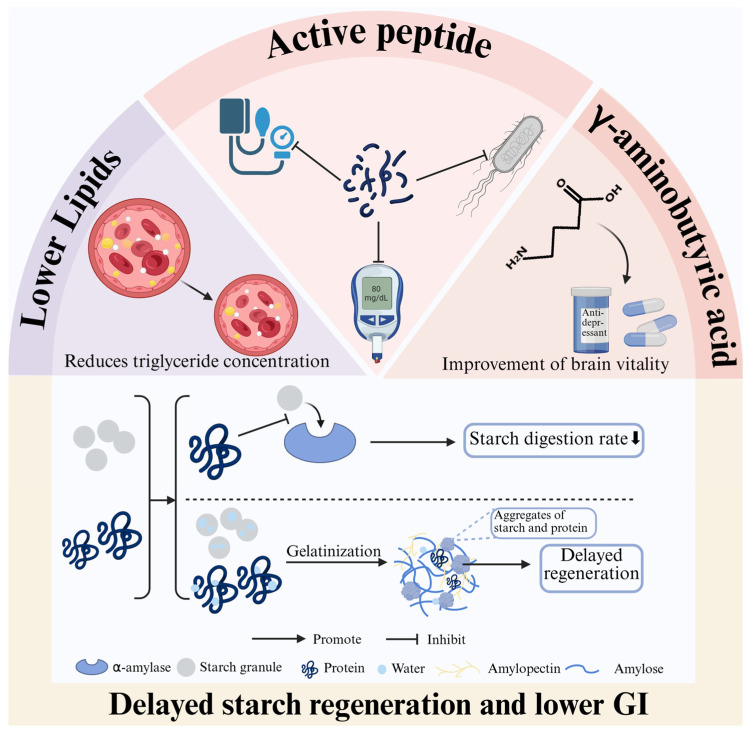
Biofunctional properties of highland barley protein.

The rich amino acid composition and unique protein structure of highland barley contribute significantly to its functional properties (Figure 1). From a biofunctional perspective, highland barley protein has been shown to reduce serum triglyceride levels and promote lipid metabolism, thereby lowering the risk of atherosclerosis and cardiovascular diseases [4]. Enzymatic hydrolysis of highland barley protein generates a variety of bioactive peptides. These peptides exhibit important physiological functions and nutritional value, particularly in lowering blood pressure and regulating blood sugar levels. Highland barley protein also contains antimicrobial peptides (barleycin) that effectively inhibit the growth of *Escherichia coli*, making them promising candidates for use as natural food preservatives [38]. In addition, highland barley is rich in γ-aminobutyric acid (GABA), a non-protein amino acid internationally recognised for enhancing brain activity. GABA is a key component in many antidepressant drugs and plays a critical role in regulating various physiological functions [39].

It was shown that highland barley proteins have a delaying effect on the short-term regeneration of highland barley starch. At the stage of starch pasting, proteins are able to build a reticular structure [40]. On the one hand it hinders the excessive swelling of starch granules and on the other hand it promotes the generation of high-density starch–protein aggregates [41]. These aggregates not only effectively inhibit the starch regeneration process but also reduce the solubilisation of amylose, thus enhancing the content of resistant starch [41]. From an enzymatic point of view, the protein significantly slows down the rate of starch digestion by masking the site of action of α-amylase [42]. This modulation of starch regeneration is important in improving the glycaemic index (GI) value of food and in combating type II diabetes [41].

In addition to the biofunctional properties described above, in terms of processing properties, the foaming capacity and foam stability of highland barley protein tend to increase with molecular weight. These properties are also influenced by the presence of sucrose, salt, and pH levels [4]. However, compared to wheat proteins, highland barley proteins contain significantly lower levels of gluten subunits, disulfide bonds, and total sulfhydryl groups. This structural limitation leads to reduced functionality in key areas such as emulsification capacity, viscosity, and gelation, thereby restricting its broader applications (Table 2) [4].

## 3. In Vitro Digestion Test of Highland Barley Proteins

### 3.1. Commonly Used In Vitro Digestive Models

In vitro digestion models are essential tools for studying protein digestion and bioavailability. These models simulate the environment of the human GI tract to assess protein digestibility. In vitro digestion models are generally classified into two types: static and dynamic systems. They range from simple, single-phase static models to complex, multicompartment dynamic systems. These models are widely used in food protein research to simulate digestive processes and quantify residual nitrogen at the end of digestion. By measuring residual nitrogen, researchers can indirectly evaluate protein digestibility [13,43].

#### 3.1.1. Static Digestion Model

Static digestion modelling typically involves establishing single digestion conditions (temperature, enzymes, pH, and bile salts) in different digestion zones and simulating the physicochemical processes involved through a series of agitation (e.g., shaking in a water bath and rotating in an air bath) [44].

Static digestion models are typified by the in vitro digestion model developed by the INFOGEST International Consortium in 2014. The model simulates the digestive conditions in the three digestive tract regions most important for food digestion: the mouth, stomach, and small intestine [45]. At each stage, a digestive solution is simulated, which includes different concentrations of various proteases and electrolyte solutions. Later, Brodkorb et al. [46] optimised the model and developed the INFOGEST 2.0 scheme. The model was optimised for INFOGEST 1.0 by including concentration specifications for CaCl_2_ in gastric and intestinal fluids and emphasising the standardisation of enzyme activity (including units) and bile assays. This recommendation greatly improves the accuracy and reproducibility of the digestion process and avoids bias in experimental results due to different enzyme activity measurement procedures.

**Figure 2:**
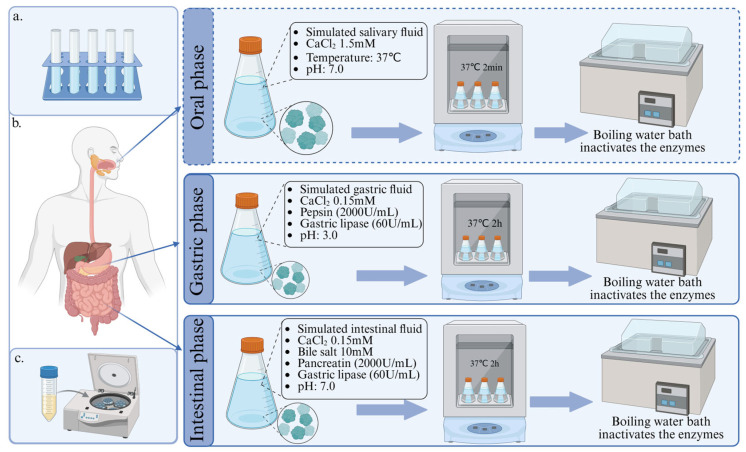
Protocols used to evaluate digestibility in the context of processed highland barley. (**a**) Determination of enzyme activity and bile assay; (**b**) oral, gastric, and intestinal digestion simulation; (**c**) centrifugation of samples to obtain supernatant.

In the case of the INFOGEST programme, for example, the static in vitro digestion model offers significant advantages due to its high degree of standardisation and low cost. These features make the model robust, reproducible, and accessible, allowing for a consistent assessment at each stage of digestion [45,46]. This static model has been widely used in the food industry over the past decade and has proved to be very useful in predicting in vivo digestive outcomes [47]. Therefore, this INFOGEST 2.0 model is the most suitable for in vitro digestion of highland barley proteins under the current conditions, and the specific scheme is shown in Figure 2. In addition, it is worth mentioning that, according to the characteristics of highland barley protein, the simulation of the oral part can be taken according to the experimental conditions, because amylase does not affect the digestion of highland barley protein, and the digestion time is short [48].

Furthermore, emerging microfluidic chip platforms are being developed. These systems utilise 3D printing or soft lithography to fabricate structural substrates, enabling dynamic cell perfusion through microfluidic mechanisms and supporting in vitro co-culture of cellular and biomaterial components [49,50]. The bioengineered chips feature transparent polymer-based microchannel networks that anatomically replicate gastrointestinal compartments (stomach, small intestine, and large intestine) [44]. The ability to test multiple conditions and factors at the same time is beneficial for single-factor experiments investigating the effect of different processing methods on the in vitro digestibility of highland barley proteins and allows for real-time monitoring of the data, which compensates to some extent for the limitations of static modelling [48].

Despite its usefulness, the static in vitro digestion model faces some limitations. One of the key issues is the over-standardisation of digestion parameters such as fixed pH, digestion duration, enzyme concentration, stirring speed, and sample size [12]. Another major limitation is the inability of static models to fully replicate the complexity of human digestion and nutrient absorption. Factors such as gastric emptying rate, dynamic enzyme activity, and nutrient transport mechanisms, which are critical for accurate estimation of digestibility, are not adequately modelled in in vitro models [51].

#### 3.1.2. Dynamic Digestive Modelling

The core objective of the Dynamic Digestion Model is to accurately simulate the dynamics of physiological processes within the digestive system, focusing on changes in pH, enzyme activity, and bile salt concentration, as well as changes in key elements such as peristaltic and emptying mechanisms of the gastrointestinal tract, and microbial inoculation, to provide a simulation that is closer to physiology [52]. Although the simulation accuracy of such models is significantly better than that of static methods, their complex operating procedures and high time costs may limit their application, and they are therefore less frequently applied to highland barley protein digestibility testing [13]. In addition, some new in vitro digestive models have been gradually applied, such as cellular models and organoid models, which are based on cell culture and simulate physiological processes through human or animal cells, which are closer to the real environment of the human body. The characteristics and application scope of these models are different, and the specific comparison is as Table 3.

### 3.2. Key Factors in the Digestive System Affecting In Vitro Digestion of Highland Barley Proteins

In in vitro digestion simulation, digestion temperature, digestion time, and digestive enzyme activity are the three core elements that determine digestion efficiency. In terms of temperature control, most experimental models use a constant temperature of 37 °C to simulate the actual digestive environment of the human body, which is based on the physiological basis of normal human body temperature. As for digestion time, it is dynamically adjusted according to the physicochemical properties of the sample, especially as the size of the food particles has a direct impact on the gastric emptying rate when the particle diameter is more than 1 mm, the gastric retention time is prolonged due to the mechanical obstruction of the pyloric valve. As a result, foods containing large particles need to be incubated in the stomach for a longer period of time. Conventional in vitro models are usually set up with a 120 min digestion cycle for each of the gastric and intestinal phases [11]. It is worth noting that when the protein concentration in the material to be measured is significantly elevated, it is necessary to extend the digestion time in parallel to ensure complete digestion while keeping other digestion parameters constant [59].

In constructing in vitro digestive models of the GI tract, the core bioactive components mainly include digestive enzyme systems (pepsin, pancreatic enzymes, trypsin, pancreatic rennet, peptidases, α-amylase, and lipase), bile salts, and mucins [11]. The enzyme activity profile in this system plays a decisive role in the simulation effect. The study showed that although some experiments used human-sourced enzyme preparations, the mainstream protocols still used omnivorous animals such as pigs and rats or human donor samples as the main enzyme source. It is worth noting that the addition of digestive enzymes follows a digestive sequence: salivary α-amylase in the oral phase, and pepsin and trypsin in the GI phase to accurately simulate the characteristics of each digestive phase [44]. Enzyme selection significantly affects the accuracy of results when assessing IVPD [60]. The research showed that the three-enzyme (trypsin, chymotrypsin and peptidase) one-step digestion could increase the degree of protein hydrolysis by 39–66% compared with the two-enzyme (pepsin and pancreatin) two-step digestion method, and the increase was closely related to the characteristics of the substrate and the assay method. This multi-enzyme synergistic system (saliva, gastric fluid, duodenal fluid, or bile components) provides higher experimental reproducibility and physiological simulation than single enzyme digestion systems.

### 3.3. Digestive Characteristics of Highland Barley Protein

Huang et al. [22] applied the INFOGEST 2.0 protocol to study the in vitro digestion of highland barley protein and found that the digested highland barley protein contained 244.06 mg g^−1^ of essential amino acids, accounting for 28.04% of the total amino acids. Among them, Leu, Phe, and Val were the most abundant essential amino acids, and the non-essential amino acids (NEAA) in highland barley protein digest were 626.34 mg g^−1^, accounting for 71.96% of all amino acids, and Pro and Glu were the major NEAA. In conclusion, after in vitro digestion, the percentage of each amino acid in highland barley protein was almost unchanged, but the content was changed [22].

The molecular weight of highland barley protein prepared by the static digestion model was hydrolysed from 10–95 kDa to a relative molecular weight of about 10 kDa. The relative molecular weights of the highland barley protein after gastric digestion were about 26–34 kDa and 10–17 kDa. After intestinal digestion, highland barley protein mimics the relative molecular mass of the gastrointestinal digest, which is approximately 10 kDa. These <1000 Da peptides are more readily absorbed than free amino acids and intact proteins [61]. After gastrointestinal digestion, the IVPD of highland barley protein was about 90.02 ± 2.24% [22].

In addition, Bohn et al. [43] emphasised that because of limited endogenous secretions, data generated by static in vitro models are usually comparable to actual digestibility results and are therefore valuable for nutritional assessment. However, some researchers have argued that IVPD values may overestimate the true nutritional value because the model does not take into account the bioavailable amino acids in highland barley [62].

## 4. Application of Highland Barley Protein in Winemaking

Highland barley protein is widely utilised in winemaking due to its rich amino acid profile and favourable functional properties. Statistics show that approximately 37.5% of Tibetan highland barley production is used for winemaking purposes [63]. Traditional highland barley wine has a long history, dating back around 10,000 years [64]. Today, most highland barley wines are produced through industrial processes, including varieties such as highland barley wine, highland barley dry red wine, highland barley beer, and highland barley nutritional wine [64].

### 4.1. The Unique Role of Highland Barley Protein in Highland Barley Wine Production

**Figure 3:**
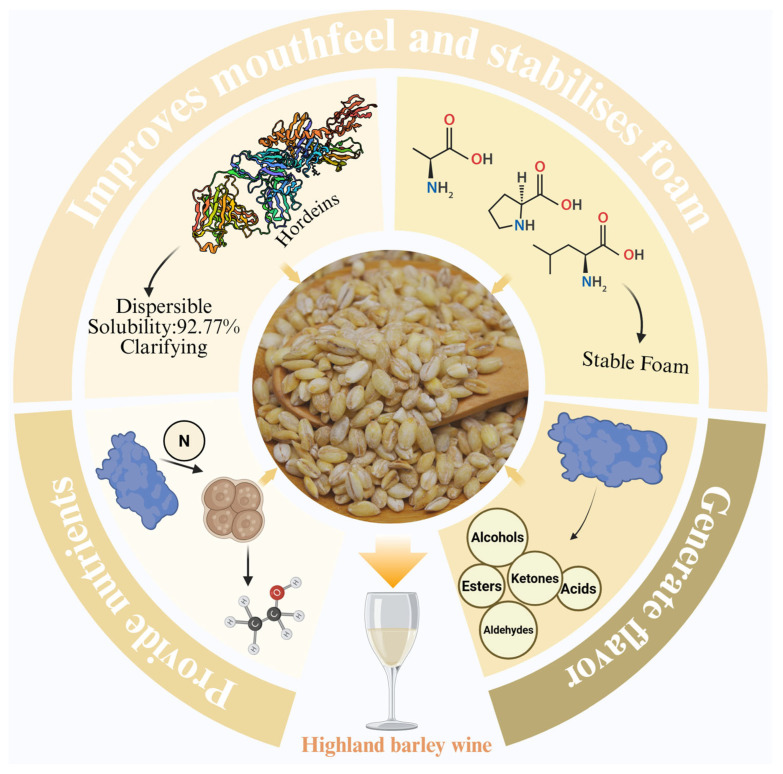
The unique role of highland barley in the production of highland barley wine.

The reason why highland barley is suitable for winemaking is inextricably linked to the characteristics of highland barley proteins (Figure 3). The high content of essential amino acids in highland barley protein increases the nutritional value of highland barley wine, which can meet the needs of the human body. It has been found that at least 17 free amino acids are found in highland barley wine [7]. At the same time, in the brewing process, the high content of highland barley protein for yeast and moulds to provide a rich source of nitrogen and a variety of growth factors, which helps the growth and reproduction of microorganisms, thus promoting their secretion of enzymes to accelerate the saccharification and fermentation process, improve fermentation efficiency. In addition, during fermentation, microorganisms secrete peptidases and further break down highland barley proteins into amino acids, and these free amino acids contribute significantly to the flavour of highland barley wine. These free amino acids are decarboxylated to form biogenic amines or precursors for the Maillard reaction, and are also degraded to aldehydes such as 4-propylbenzaldehyde, phenylacetaldehyde, and other flavour compounds through the Strecker reaction [17]. This is important for the flavour of highland barley wine.

The higher solubility of highland barley protein can be better applied to highland barley wine to make it more clarifying. The solubility of highland barley protein is affected by temperature and pH, and its solubility shows a trend of decreasing and then increasing with the increase in pH, and the solubility of highland barley protein is as high as 92.77% in the range of 45–55 °C [4]. The presence of proteins in the liquor usually causes turbidity [65]. This phenomenon mainly originates from the interaction and aggregation process of proteins with metal ions (e.g., copper ions) or polyphenols. Specifically, polyphenolic components are able to bind to protein molecules through covalent binding and non-covalent interactions to form microscopic complexes. These complexes further aggregate to form macroscopically visible precipitation particles that are eventually deposited at the bottom of the vessel [66]. However, the high content of hordeins in highland barley cleverly solves this problem. Hordeins contain high levels of hydrophobic amino acids (e.g., alanine, proline, and leucine), which are easily dispersed in the alcoholic solution and do not affect the phenolics in the wine, which have less impact on the colour and anthocyanin content of the wine and can be used as clarifiers [26,67,68]. In addition, highland barley is rich in hydrophobic amino acids, which leads to strong protein aggregation and high surface hydrophobicity, which can produce stable foam in beer [69].

### 4.2. Traditional and Modern Brewing Processing Techniques

Traditional highland barley wine brewing is often based on solid-state fermentation of highland barley as raw material; the main feature is that saccharification and fermentation are carried out at the same time [70]. The main processes are as follows. A total of 30 kg of highland barley was cooked in boiling water for 2 h to obtain a soft texture. After cooling the boiled highland barley to room temperature, the boiled highland barley was mixed with Qu (1 kg with 5 g Qu) and transferred to a quilt-wrapped wine jar to keep it warm. Fermentation was carried out at room temperature and continued in solid state for 48 h, i.e., Jiupei. Finally, sterile water was added to the fermented Jiupei and filtered to obtain a pale yellow highland barley wine [71]. Among them, the Jiuqu is a key factor in the quality and flavour of the wine, which contains bacteria, yeasts, and moulds [72].

**Figure 4:**
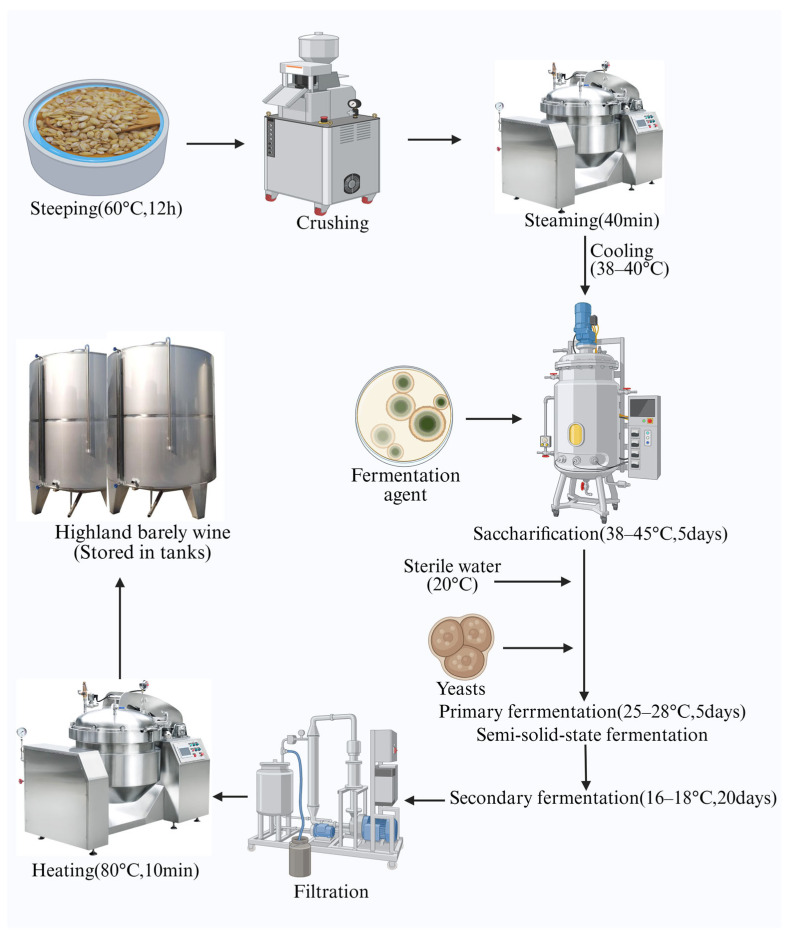
The modern brewing process of highland barley wine.

Modern brewing technology is more industrialised and mechanised, and is characterised by the separation of saccharification and fermentation [73]. The main steps described by Zhang et al. [73] are as follows (Figure 4). The highland barley was steeped in water at 60 °C for 12 h. After steeping, the highland barley was crushed, which facilitated saccharification. It was then steamed in a steamer for 40 min at a controlled time and temperature. It was then cooled to 38–40 °C. A laboratory-cultured fermentation agent (including *Aspergillus albicans*, *Aspergillus niger*, *Rhizoctonia solani*, amylase, and proteases, etc.) was added. The mixture was transferred to a stainless steel tank. It was then sugared in a tank for 5 days, maintaining a temperature of 38–45 °C. Sterile water at 20 °C (the same amount as the dried raw material) and yeast (0.1%, *w*/*w*, of the dried raw material, typically brewer’s yeast and candida) were added to the stainless steel tanks, and semi-solid fermentation was initiated for 5 days at 25–28 °C (primary fermentation). The fermentation temperature was then adjusted to 16–18 °C for secondary fermentation. The mixture was left to stand for 20 days until an alcohol content was reached for a mature highland barley wine product. The fermented mash was clarified, and the supernatant was filtered using a filtration system to remove impurities and ensure the clarity of the liquor. Finally, the filtrate was heated at 80 °C for 10 min and stored in wine storage containers [73].

In the traditional brewing process, a natural mixture of microorganisms is used as the fermentation agent. In contrast, the modern process employs laboratory-cultured moulds and yeasts. These cultured strains are safer because they are well-identified and free from contamination by unwanted bacteria. Traditional Jiuqu relies mainly on moulds to secrete protease and amylase. In this process, saccharification and fermentation occur simultaneously, which is relatively inefficient. In the modern method, saccharification and fermentation are carried out separately. Exogenous enzymes are added to hydrolyse proteins and starch, providing more readily available substrates for yeast fermentation. This significantly shortens the fermentation cycle and improves overall efficiency [73].

Despite differences between the traditional and modern methods, both processes involve three key steps: thermal processing (steaming), enzymatic treatment, and microbial fermentation. These steps alter the structure of highland barley proteins in various ways, enhancing their digestibility. This transformation is also essential for ensuring the quality of highland barley wine.

## 5. Effect of Different Processing Methods on Highland Barley Protein Digestibility in Highland Barley Wine Production

### 5.1. The Effect of Key Processing Techniques on Highland Barley Protein Digestibility During Winemaking

#### 5.1.1. Thermal Processing

Common heat treatments include roasting, boiling, and steaming, with steaming being the primary method used in winemaking. Heat treatments cause proteins to unfold, leading to the loss of their quaternary, tertiary, and secondary structures. During this process, the secondary structure shifts from β-sheets to random coils, resulting in protein denaturation [74].

Denaturation can have two opposing effects on IVPD. On one hand, unfolding exposes enzymatic cleavage sites, allowing digestive enzymes easier access and thus improving digestibility. On the other hand, excessive unfolding can lead to protein aggregation. These larger, complex structures hinder enzyme access, reduce hydrolysis efficiency, and may even trigger amino acid desulfurization and isomerization, thereby lowering digestibility and biological value [75,76].

For example, Becker et al. [29] found that thermal treatment of sorghum proteins promoted disulfide bond formation and polymerization. These disulfide cross-links stabilise the protein structure, decreasing enzyme activity in the GI tract and reducing both solubility and digestibility [77]. Both beneficial and adverse effects can occur depending on the temperature and duration of thermal processing. Therefore, it is essential to carefully control steaming conditions during highland barley processing to prevent excessive heating.

Regarding treatment types, dry heat has been found more effective than moist heat in improving digestibility. Moist heat treatments such as boiling and pressure cooking did not enhance the protein digestibility of millet, whereas roasting significantly improved its IVPD [37]. If processing conditions allow, dry heat treatments could also be explored for highland barley. This may help optimise the production process and improve protein digestibility.

In addition to the protein structure changes induced by thermal treatments, anti-nutritional factors are also degraded to some extent during thermal treatments, thereby improving protein digestibility. For example, Alexandra et al. [78] claimed that thermal treatments (15 min at 95 °C) reduced trypsin inhibitor activity in lentil protein by 86% after the study. The same finding was made by Bailey et al. [79], who subjected rapeseed isolate protein to thermal treatments (90 °C for 10 min) and found an increase in its digestibility (from 78.4% to 97.5%). This phenomenon may be related to the inactivation of trypsin inhibitors and breakage of disulfide bonds due to heat treatment, which enhances the efficiency of the action of digestive enzymes on proteins.

The cross-linking of proteins with other substances occurs during the thermal processing of food. The amino part of amino acids and the carbonyl group of reducing sugars undergo a Maillard reaction under the influence of heat, in which harmful derivatives such as methylglyoxal (MGO) and advanced glycation end-products (AGEs) are formed [55]. The reaction of MGO with glutelin in highland barley promotes a decrease in the hydrophobicity of the glutelin surface and an increase in disulfide bonds, and MGO induces strong aggregation of glutelin upon heating, leading to the masking of protease cleavage sites and a significant reduction in digestibility [80]. On the other hand, AGEs are progressively more resistant to enzymatic degradation during the glycosylation process, primarily because they resist digestive proteases by blocking trypsin cleavage sites or exhibiting spatial site resistance, thereby reducing digestibility [81]. Duodu et al. [35] found that under optimal conditions, sorghum tannins can bind and precipitate at least 12 times their weight of protein. Moreover, tannin–protein interactions in sorghum are thought to involve hydrogen bonding and nonpolar hydrophobic conjugation. Thermal processing can break hydrogen bonds, which reduces the cross-linking of phenolics to proteins [82]. Therefore, proteins can bind more effectively to digestive enzymes, thereby increasing IVPD. In addition, in some grains, starch granules are surrounded by a large number of globular proteins embedded in the protein matrix. When starch is pasted after cooking, it may compete with proteins for proteolytic enzyme binding sites, thus reducing proteolytic enzyme accessibility to the proteasomes and reducing protein digestibility [35].

#### 5.1.2. Enzymatic Treatment

The addition of exogenous enzymes can effectively improve protein digestibility, mainly including phytase and protein hydrolase. The addition of food-grade phytase can gradually degrade phytic acid, thereby affecting the properties of the phytic acid–protein complex and making proteins easier to digest [83]. For example, Rosa-Sibakov and Natalia et al. [83] found that when fava bean meal was treated with a high dose of phytase (20 U), the phytic acid content was reduced by more than 80%, while the free amino nitrogen content was at its highest level. Protein hydrolysis enzymes (both proteases and peptidases) play a key role in this process: proteases break down large proteins into small peptide fragments, while peptidases further hydrolyse specific peptide bonds or degrade peptide fragments completely into amino acids, thus significantly improving the digestibility and absorption of the final product [84].

Improvement in protein digestibility in vitro was more pronounced with the complex enzyme than with the enzyme alone [76]. For example, Wang et al. [76] found that egg white proteins treated with a combination of pepsin and trypsin contained more low molecular weight peptides with higher signal intensities (*m*/*z* < 849.2) compared to pepsin-treated egg white proteins. The number of unique peptides identified in each digestion product was positively correlated with its in vitro digestibility, suggesting that the combination enzymes may better enhance IVPD.

#### 5.1.3. Fermentation

In terms of structure, microbial fermentation affects protein catabolism and bioavailability mainly by promoting three phases of primary hydrolysis, secondary hydrolysis, and free amino acid catabolism of highland barley protein [85]. Firstly, the microorganisms during fermentation produce organic acids that lower the pH of the environment, which activates the primary activity of the cereal proteases. At the same time, reduction of disulfide bonds occurs, and intramolecular disulfide bonds are converted to intermolecular forms. Both together lead to primary protein hydrolysis. The multilevel structure of the protein is altered, becoming looser and allowing the release of polypeptides of various sizes [86]. Then, the intracellular peptidases of the microorganisms undergo secondary protein hydrolysis to release free amino acids [85]. This state is more conducive to their efficient hydrolysis during digestion and improves digestibility. Finally, microorganisms perform the same various catabolic reactions on free amino acids to degrade them [87]. In conclusion, microbial fermentation increases the peptide and amino acid content and significantly improves the amino acid profile (higher content of essential amino acids), which ultimately improves the bioavailability and digestibility of proteins [85]. In addition, for special populations (allergic to grains), changes in protein and peptide structure also increase the biological activity of the product, altering its allergenicity and improving digestibility [88].

Fermentation also plays a significant role in the degradation of anti-nutritional factors. This is mainly due to the build-up of organic acids during fermentation, which lowers the pH and provides favourable conditions for the endogenous phytase of the grains (optimal pH around 5.0). Phytase hydrolyses phytic acid and produces a variety of phosphates and myoinositols. These compounds have a low chelating capacity, which increases the bioavailability of proteins, peptides, and amino acids [87].

### 5.2. Comparative Analysis of Different Processing Methods

**Figure 5:**
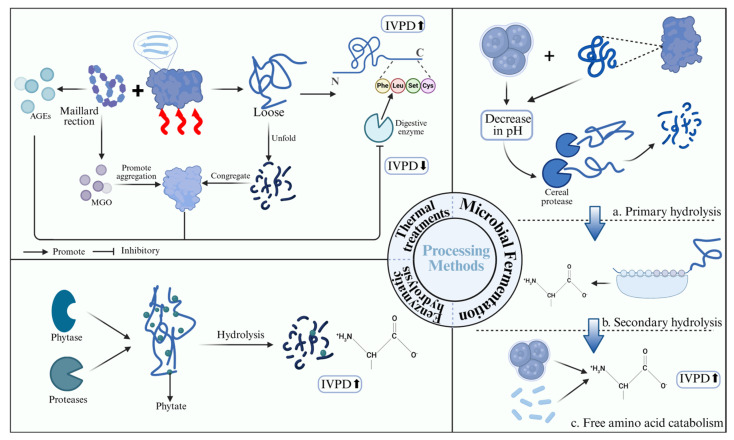
Effect of processing methods of thermal processing, fermentation, and enzymatic treatment on protein structure and in vitro protein digestibility.

In summary, thermal treatments, microbial fermentation, and enzymatic hydrolysis improve protein digestibility through two main mechanisms. First, they induce structural modifications in proteins, including depolymerization and changes in secondary and tertiary structures. Second, they influence protein interactions with anti-nutritional factors, phenolics, reducing sugars, and starch. Among these, the Maillard reaction between proteins and reducing sugars plays a key role in altering digestibility (Figure 5).

Although different processing methods alter the kinetics of protein digestion and absorption, the various processing methods differ and have their own characteristics [88]. In terms of protein conformational changes, thermal treatment usually denatures proteins by breaking hydrogen bonds at high temperatures, causing proteins to lose secondary, tertiary, or even quaternary structure, which becomes looser and exposes enzyme-binding sites, thus making them easier to bind to digestive enzymes, but leaving the primary structure intact. Enzymatic hydrolysis, on the other hand, breaks down large proteins into small peptides and amino acids by hydrolysis of peptide bonds, directly destroying the primary structure of proteins and lowering their molecular weight, which is a complete destruction of protein conformation. Fermentation is also essentially enzymatic hydrolysis, in which microorganisms indirectly hydrolyse proteins by reducing pH and producing proteases. Therefore, enzymatic hydrolysis and fermentation are more effective in breaking down proteins based on the mechanism, which results in a deeper change in digestibility. In terms of digestibility change, thermal treatments have a duality. Moderate thermal treatments can improve digestibility, but overheating can have a negative effect, so it is necessary to control the processing method and temperature.

In other respects, thermal treatments involve only heat and moisture, focusing mostly on their role in the green processing of food. However, excessive thermal processing can also produce some harmful by-products. Amino acids and reducing sugar undergo a Maillard reaction under thermal treatments, and in the final stages of this reaction, large amounts of harmful derivatives such as dietary advanced glycosylation end-products (dAGEs), methylglyoxal (MGO), heterocyclic aromatic amines (HAAs), and acrylamide (AA) are formed [55]. Research has shown that these toxic compounds are often involved in oxidative stress, DNA damage, inflammation, and cell-damaging functions that lead to the development of cancer and other chronic diseases [89]. Enzymatic hydrolysis can produce some functionally active peptides for antioxidants, blood pressure, and blood sugar reduction. However, compared with large peptides, small peptides are difficult to fully unfold and rearrange at the interface due to structural limitations, resulting in their weaker ability to reduce interfacial tension and stabilise emulsions/foams [90]. Enzymatic hydrolysis also produces bitter peptides that affect the flavour of the food, and the high cost of enzymes may make it difficult to put them into large-scale production for the food industry [91]. Fermentation differs from enzymatic hydrolysis in that fermentation produces a series of secondary metabolites such as peptides, oligosaccharides, vitamins, and polyphenols, as well as volatile flavouring substances such as esters and aldehydes, which greatly enhance the nutritional value and flavour of foods [92].

### 5.3. The Significance of Increased Protein Digestibility for Highland Barley Wine Production

The improvement of highland barley protein digestibility after processing is important in the production of highland barley wine, mainly in the enhancement of its nutritional value, the improvement of sensory characteristics, and the optimisation of the processing process. In terms of nutritional value enhancement, compared with the original highland barley grain, the proportion of essential amino acids in the amino acid of highland barley wine after microbial fermentation increased, probably because of the conversion of NEAAinto essential amino acids in the brewing process [63]. At the same time, increased digestibility indicates the production of more functional and active peptides, including dipeptidyl peptidase inhibitory peptides, antiplatelet peptides, and antibacterial peptides (barleycin) [4]. They are important in lowering blood sugar concentration, inhibiting bacteria and microorganisms, and inhibiting platelet aggregation [4]. In addition, the breakdown of proteins reduces their cross-linking with starch and phenols, releasing more free phenols (e.g., ferulic, p-coumaric, and caffeic acids) and sugars, which can improve the antioxidant activity as well as the alcoholic strength of highland barley wine [93].

In terms of organoleptic quality, the hydrolysis of large protein molecules into smaller peptides and amino acids during processing enhances protein–water interactions. These smaller protein particles allow for better hydration of peptide and amino acid side chains, improving solubility and resulting in a clearer appearance of highland barley wine [94].

During fermentation, microbial activity also alters the structural characteristics of highland barley. It increases porosity and specific surface area, which enhances the interaction between capillary water and the highland barley matrix while reducing the mobility of water. As a result, the hardness of highland barley decreases by 20.43–58.60% after fermentation. This reduction is mainly attributed to cellulase secreted by microorganisms, which hydrolyses cellulose and significantly lowers chewiness, elasticity, and gumminess—contributing to a smoother mouthfeel of the final product [85].

Additionally, the interaction between macromolecular proteins and polyphenols—often responsible for aggregation and turbidity—is weakened after processing. This reduction in cross-linking helps improve the clarity of highland barley wine [66].

From a process perspective, the improved digestibility of highland barley protein enables more rapid breakdown into small peptides and amino acids. These breakdown products serve as accessible nitrogen sources for fermenting microorganisms. As a result, the time required for microbial degradation of large proteins is reduced, ultimately enhancing fermentation efficiency.

## 6. Conclusions and Prospects

The key processing methods involved in the processing of highland barley proteins for winemaking include thermal processing, enzymatic treatment, and microbial fermentation. These methods achieve efficient utilisation of highland barley proteins by significantly improving protein digestibility by altering the structure of the protein and reducing or removing the interaction of other substances with the protein. Thermal processing loosens the structure of highland barley proteins, exposing enzyme binding sites and thus making it easier to bind to digestive enzymes. Enzymatic treatment, on the other hand, breaks down large proteins into small peptides and amino acids by hydrolysing peptide bonds, reducing molecular weight, and destroying protein conformation. During microbial fermentation, highland barley protein provides a nitrogen source for microbial metabolism and promotes its growth and metabolism, thus degrading anti-nutritional factors such as phytic acid and tannins, and producing proteases, amylases, cellulases, and other enzymes to hydrolyse proteins and produce amino acids and peptides. This process not only improves the nutritional value of highland barley wine but also improves the taste and acceptance of highland barley wine. In addition, the functional active peptides produced by the hydrolysis of highland barley protein play an important role in lowering blood pressure and sugar, and in the future, we can apply these processing methods to explore the in-depth development of highland barley in the field of winemaking and the development of other functional foods, so as to achieve the efficient and comprehensive use of highland barley protein resources. In terms of in vitro protein digestibility testing, due to the limitation of conditions, the current measurement is a more static in vitro model, and lacks the application of a dynamic system. Moreover, nowadays, the research on the dynamic system is mostly confined to the simulation of individual organs, such as the stomach and intestines, but there is no systematic simulation of the whole process. Therefore, in protein digestibility testing, a dynamic in vitro digestion model can be constructed in the future to reveal the proteolytic pathway in the digestion process. Further, we can explore the in vivo digestion of proteins and compare it with in vitro digestion to continuously improve the in vitro digestion model.

## Figures and Tables

**Table 1:** A comparison of the amino acid profile of highland barley with other cereal proteins (mg/g DW).

Amino Acid Type		Highland Barley [19]	Barley [20]	Wheat [21]	Oat [21]
Essential amino acid	Methionine (Met)	1.52	0.5	1.9	2.8
	Lysine (Lys)	3.64	3.9	3.6	4.4
	Valine (Val)	4.66	0.9	5.7	6.1
	Isoleucine (Ile)	3.05	3.8	5.1	4.5
	Phenylalanine (Phe)	4.66	5.5	6.6	6.5
	Leucine (Leu)	6.32	7.4	8.2	9.4
	Threonine (Thr)	3.26	4.0	3.7	4.2
	Overall amount	27.11	26.0	28.8	37.9
Non-essential amino acid	Asparaginic acid (Asp)	5.87	5.9	7.0	8.7
	Cystine (Cys)	1.80	5.5	2.2	4.4
	Proline (Pro)	9.73	9.8	12.8	7.7
	Arginine (Arg)	5.26	5.6	6.5	9.7
	Glutamic acid (Glu)	20.71	26.5	29.4	25.7
	Tyrosine (Tyr)	2.77	3.3	2.7	3.6
	Serine (Ser)	3.95	4.6	6.3	5.3
	Glycine (Gly)	3.95	4.6	5.2	5.8
	Alanine (Ala)	4.23	4.6	4.7	5.2
	Histidine (His)	2.08	1.2	2.5	3.3

Note: Tryptophan (Trp) was destroyed in hydrolysis and could not be determined. DW: Dry weight.

**Table 2:** Composition and characterisation of highland barley proteins.

Protein Type	Percentage	Molecular Weight (SDS-PAGE Analysis)	Structural Characteristic	Key Amino Acids and Compositional Characteristics	Functional Characteristics
albumins	12.95%	12–60 kDa	Ductile, rough, porous surface, loose texture	high contents of Lys (the main limiting amino acid in the body), Trp, and Met	Its water solubility allows it to compete with starch for more water molecules.
globulins	12.73%	12–60 kDa	Mainly comprises 7S- and 11S-globulins	high contents of Asp, Glu, Arg, Leu, and Lys	Total water uptake by starch granules can be reduced during the highland barley starch pasting process.
hordeins	16.96%	30–70 kDa	The main secondary structure is β-folding and β-turning, which is conducive to the formation of a compact globular protein structure and more stable structure.	high contents of Glu, Pro, Trp, and Leu; high contents of hydrophobic amino acids; low contents of essential amino acids	It supplies carbon and hydrogen to highland barley seeds and has a higher emulsion stability index; better stabilisation of oil droplets; higher thermal stability than maize hordeins; and lower surface hydrophobicity.
glutelins	47.83%	40–300 kDa	The secondary structure was mainly dominated by β-folding; the contents of disulfide bonds and total sulfhydryl groups were 10.3779 μmol/g and 88.2799 μmol/g, respectively, much lower than wheat glutelins.	rich in Glu and Pro, with 34.35% essential/total amino acid content	It has high thermal stability, which is unfavourable for moisture absorption and partial spreading; its hydrate is adhesive and elastic, which provides strength and elasticity to the dough.

**Table 3:** Advances in in vitro digestive modelling.

Model Type	Representative Model	Simulation of Digestive Phase	Main Features	Scope of Application	References
Static model	INFOGEST	Mouth, stomach, small intestine	Temperature, incubation time, forces, pH, mineral activity, enzyme activity, mucin levels, and bile salt levels were specified for each digestive area; saliva, gastric, and small intestinal fluids were standardised.	Used to measure the final concentration of commonly digested products (e.g., peptides, amino acids, fatty acids, and sugars) after a product has passed through the GI tract, as well as the rate and extent of digestion of a large number of nutrients over time.	[45]
	Microfluidic chip model	Stomach, intestines	It features a network of transparent polymer microchannels that mimic gastrointestinal tract compartmentalisation, each lined with cells such as intestinal epithelial cells and vascular endothelial cells. A sophisticated pump-valve configuration adjusts hydrodynamic parameters to simulate peristaltic and digestive secretion patterns. Small size.	Real-time monitoring and online analysis of food data in the GI tract.	[44]
Dynamic modelling	DGM (Dynamic Gastric Modelling)	Stomach	It is a very close reproduction of dynamic conditions based on monogastric animals and the human interior, accurate for gastrointestinal transit, pH, bile salt concentration, and glucose absorption data.	Study of the fate of ingested components (e.g., food, microorganisms, and drugs) during gastrointestinal transport.	[53]
	HGS (Human Gastric stomach)	Stomach	It simulates the continuous peristalsis of the gastric wall with contractile forces similar in amplitude and frequency to those reported in vivo, and incorporates gastric secretion, emptying systems, and temperature control.	This approach allows for a comprehensive assessment of changes in GI content characteristics through the evaluation of physicochemical parameters, as well as the study of the modulation of digestive matrix breakdown and release of bioactive compounds by biological factors, including changes in pH, patterns of enzyme activity, and muscular motility mechanisms.	[54]
	TNO Gastrointestinal tract model (TIM-1)	Stomach, small intestine	Dynamic pH curve fitting, peristaltic mixing, addition of bile and acetyl digestive enzymes, and passive absorption.	For modelling the digestive behaviour of food and drugs in the GI tract with good predictive power.	[55]
	SHIME (Simulator of the Human Intestinal Microbial Ecosystem)	Stomach, small intestine	More realistic simulation of microorganisms in the GI tract; multi-compartment design allows simulation and integration of the entire GI tract; real-time monitoring device allows continuous measurement of parameters such as pH, temperature, and so on.	Modelling food digestion and absorption processes, studying nutrient metabolic pathways, and assessing their impact on the gut microbiota.	[56]
New model	Caco-2 cell model	Intestinal tract	Simulation of transmembrane transport and bioavailability of digestive products by intestinal epithelial cells; direct reflection of intestinal absorption potential.	Transport, absorption, and permeability of substances in the gut and the effect of drug dosage forms, precursors, carriers, and structures on absorption.	[57]
	Organoid models	Intestinal tract	Organoid derived from adult stem cells mimics the human digestive system.	Complex food digestion and absorption, a study on the interaction with human immunity.	[58]

## Data Availability

No new data were created or analysed in this study.
